# Association of Air Pollution with the Number of Common Respiratory Visits in Children in a Heavily Polluted Central City, China

**DOI:** 10.3390/toxics11100815

**Published:** 2023-09-28

**Authors:** Dan Wang, Yanan Wang, Qianqian Liu, Wenxin Sun, Liangkui Wei, Chengxin Ye, Rencheng Zhu

**Affiliations:** 1 Emergency Department, The Third Affiliated Hospital of Zhengzhou University, Zhengzhou 450014, China; wangd8723@126.com (D.W.); liuqian2899@126.com (Q.L.); yecx01@126.com (C.Y.); 2 Emergency Department, Maternal and Child Care Service Centre of Henan, Zhengzhou 450014, China; 3 School of Ecology and Environment, Zhengzhou University, Zhengzhou 450001, China; wangyn1175@163.com (Y.W.); sunwx123@gs.zzu.edu.cn (W.S.)

**Keywords:** air pollutants, respiratory diseases, generalized additive model, meteorological factors

## Abstract

Children’s respiratory health is vulnerable to air pollution. Based on data collected from June 2019 to June 2022 at a children’s hospital in Zhengzhou, China, this study utilized Spearman correlation analysis and a generalized additive model (GAM) to examine the relationship between daily visits for common respiratory issues in children and air pollutant concentrations. Results show that the number of upper respiratory tract infection (URTI), pneumonia (PNMN), bronchitis (BCT), and bronchiolitis (BCLT) visits in children showed a positive correlation with PM_2.5_, PM_10_, NO_2_, SO_2_, and CO while exhibiting a negative correlation with temperature and relative humidity. The highest increases in PNMN visits in children were observed at lag 07 for NO_2_, SO_2_, and CO. A rise of 10 μg/m^3^ in NO_2_, 1 μg/m^3^ in SO_2_, and 0.1 mg/m^3^ in CO corresponded to an increase of 9.7%, 2.91%, and 5.16% in PNMN visits, respectively. The effects of air pollutants on the number of BCT and BCLT visits were more pronounced in boys compared to girls, whereas no significant differences were observed in the number of URTI and PNMN visits based on sex. Overall, air pollutants significantly affect the prevalence of respiratory diseases in children, and it is crucial to improve air quality to protect the children’s respiratory health.

## 1. Introduction

In the wake of ongoing economic and industrial advancement, air quality has attracted increasing attention from people. Notwithstanding the diligent efforts exerted by nearly all nations to mitigate air pollution, it persists as a salient and intractable predicament that demands sustained consideration. More than 80% of the world’s urban population lives in areas where air quality exceeds World Health Organization (WHO) limits [1], and environmental air pollution caused an estimated 4.2 million premature deaths worldwide in 2019 [2], highlighting the pressing and consequential issue of health problems caused by air pollution [3]. In general, air pollution is one of the most important causes of human health [4], affecting metabolic capacity [5] as well as the health of the respiratory [6,7], circulatory, nervous, digestive, and urinary systems [8,9,10,11]. Specifically, PM_2.5_ can penetrate deeper into human tissues, leading to serious diseases such as dermatitis, conjunctivitis, and even lung cancer [12,13], and its adverse effects cause a welfare loss of 1388.4 USD/person/year [14]. There is a significant relationship between PM_10_ and male lung cancer incidence [15], with elevated concentrations of PM_10_ posing an increased risk of allergies among populations [16]. In addition, NO_2_ has a significant correlation with cardiovascular mortality [17], while SO_2_ can lead to type 2 diabetes [18]. Thus, there have been calls for attention to the need to reduce potential risks to human health from air pollution [19]. Research indicates that the respiratory system exhibits the highest sensitivity to fluctuations in air quality [20]. Due to the incomplete development of functions such as immunity and metabolism [21], children are more susceptible to the adverse effects of air pollution on their respiratory health [22,23]. According to statistics, 25.7% of hospitalized children in China are admitted due to respiratory diseases [24], such as upper respiratory tract infections (URTIs), pneumonia (PNMN), bronchitis (BCT), and bronchiolitis (BCLT). Early exposure to air pollution can lead to impaired lung function and increase the risk of children developing respiratory diseases [25]. Nevertheless, the majority of child fatalities attributed to respiratory diseases can be avoided with proactive prevention measures [26]. Therefore, it is imperative to study the effects of air pollutant concentrations on the number of common respiratory visits in children, which can establish a theoretical basis for protecting children’s health and mitigating air pollution.

Currently, studies on how air pollution affects the incidence of respiratory disease patient visits are sufficient [27,28,29,30,31], but most studies have focused on the overall prevalence of respiratory diseases in children [32,33,34,35,36]. Fewer studies have explored the relationship between the number of different respiratory disease visits in children and the concentration of air pollutants. Due to the varying locations of different respiratory diseases within the respiratory system, theoretically, there should be differences in the impact of air pollutants on these diseases. The effect of air pollution on the number of common respiratory visits in children is understudied in terms of sex and age classification, and studies on less severe air pollution are not applicable to areas with severe air pollution. Hence, it is essential to conduct detailed research on the correlation between pediatric respiratory disease visits, categorized by different diseases, gender, and age, and air pollutant concentrations in regions with severe air pollution, thus contributing to the existing gap.

Perceptibly, air pollution has become one of the most typical environmental concerns in Central China, especially in the city of Zhengzhou in Henan Province. The complex air pollution situation poses a significant threat to the respiratory health of people, particularly children. Thus, this study investigated the association of air pollutants with the number of visits for four common respiratory diseases in children. The specific objectives were (1) to analyze the correlation between the number of patient visits and the concentrations of pollutants, temperature, and relative humidity; (2) to determine the impact of single pollutants on the number of patient visits at different lag days; (3) to investigate the influence of pollutants on the number of patient visits for different sex and age groups; and (4) to analyze the impact of two pollutants on the number of patient visits.

## 2. Materials and Methods

### 2.1. Source of Data

The consultation data for children with four common respiratory diseases were extracted from the outpatient HIS system of the Third Affiliated Hospital of Zhengzhou University (and Henan Provincial Maternal and Child Health Hospital). The respiratory department of this hospital has a broad range of case sources, covering almost one third of children with respiratory diseases in Zhengzhou. Therefore, the consultation data are highly representative. For this study, we collected information on the respiratory disease visits of patients from 1 June 2019 to 30 June 2022. The information includes the date of the visit, sex, age, and the type of disease, which covered upper respiratory tract infections (URTI, J06.900x003), pneumonia (PNMN, J18.901), bronchitis (BCT, J40.x00), and bronchiolitis (BCLT, J21.900).

The air quality data were obtained from the China Air Quality Platform (https://www.aqistudy.cn/historydata/about.php (accessed on 1 November 2022)). We selected air quality index (AQI) and pollutant detection data comprising PM_2.5_, PM_10_, SO_2_, NO_2_, O_3_-8h, and CO levels in Zhengzhou.

The temperature and relative humidity data were obtained from the rp5.ru platform (https://rp5.ru (accessed on 15 September 2022)). The platform provides data every three hours; thus, the average of the eight daily data points was considered as the meteorological daily average.

### 2.2. Methods

#### 2.2.1. Processing of Data

During the study period, extensive social control measures were implemented in Zhengzhou in response to the outbreak of COVID-19 and severe flooding. As a result, restrictions on travel during this period influenced the attendance of patients with minor illnesses. The concentrations of air pollutants during the closure period were reduced compared to normal periods; for example, the daily concentration of PM_10_ decreased by 77% [37]. Based on preliminary analysis, it was found that the number of common respiratory visits for children during the control period showed a weak correlation with air pollutants. To obtain more accurate conclusions, data from the control period were excluded from this study to minimize disturbances. Data for the following periods were excluded: 5 February 2020 to 10 May 2020; 24 February 2021 to 7 March 2021; 20 July 2021 to 24 July 2021; 4 August 2021 to 10 September 2021; and 22 January 2022 to 20 February 2022.

Consultation data were further disaggregated by sex and age groups, adopting Xu et al.’s [38] methodology, which grouped children’s cases by ages: <1 year, 1–3 years, 4–6 years, 7–13 years, and 14–16 years old.

#### 2.2.2. Spearman Correlation Analysis

Spearman correlation analysis was used to study the correlations between the number of respiratory visits in children, air pollutants, temperature, and relative humidity. The correlation coefficient “r” was used to evaluate the correlation between the factors, as a non-parametric indicator of the dependence of two variables.

#### 2.2.3. Generalized Additive Model (GAM)

The GAM is widely used to analyze time series data on environmental and health issues. The GAM, which is based on a Poisson distribution, was used to analyze the effect of air pollutants on the number of pediatric respiratory visits. Temperature, relative humidity, long-term trends over time, and the “day of the week” effect were included in the model, taking into account studies [34,36] on air pollution and human health. Consideration of these variables, which could affect either the number of visits or the concentration of air pollutants, resulted in a more accurate correlation between the number of respiratory visits in children and the concentration of air pollutants. The model expression is

Log [E(Y_t_)] = α × X_t_ + s(T, df) + s(RH, df) + s(time, df) + as factor(DOW) + β (1)

where Y_t_ is the number of URTI, PNMN, BCT, and BCLT visits in children on observation day t; α is the model coefficient; X_t_ is the air pollutant concentration on observation day t in μg/m^3^ (with CO concentration in mg/m^3^); s is the spline function for T, RH, and time; T is the temperature on the observation day given in degrees Celsius; df is the degrees of freedom; RH is the relative humidity on the day of observation; “time” represents daily time and is a series indicating the order of occurrence of respiratory visits in children in this study, which takes the values 1, 2, 3, etc.; DOW is the weekly dummy variable; and β is the intercept.

The research conducted a quantitative analysis of the impact of air pollutants on the number of common respiratory visits in children using Excess Risk (ER). ER represents the change in the percentage of the number of visits per unit increase in pollutant concentration. The considered air pollutants included PM_2.5_, PM_10_, NO_2_, and O_3_-8h, with increases in concentration of 10 μg/m^3^, and SO_2_, with an increase in concentration of 1 μg/m^3^. In addition, CO was considered with an increase in concentration of 0.1 mg/m^3^. ER and the 95% CI were calculated as follows:

ER = [exp (10 × α) − 1] × 100%(2)

95% CI = {exp [10 × (α ± 1.96SE)] − 1} × 100%(3)

where α is the GAM regression coefficient and SE is standard error.

Continuous data from 11 May 2020 to 23 February 2021 were selected after excluding the control period data and applied to the GAM to study the single-day lag (from lag 0 to lag 7) effect and the multiple-day average lag (from lag 01 to lag 07) effect.

## 3. Results and Discussion

### 3.1. Spearman Correlation Analysis

**Figure 1:**
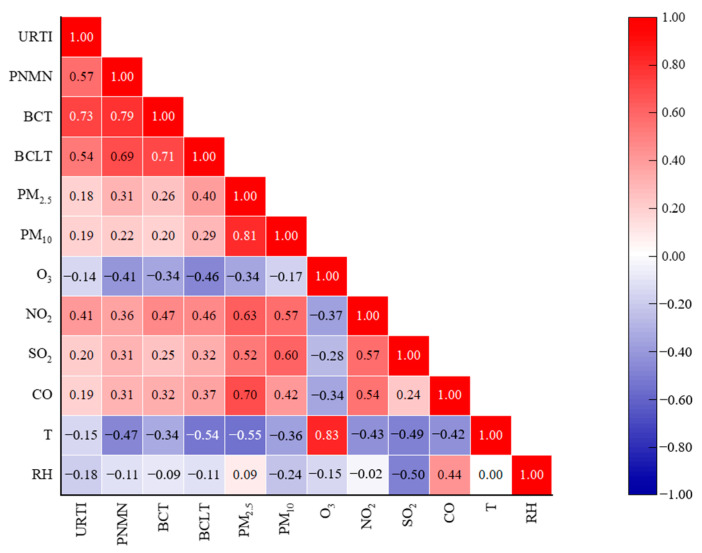
Correlation heat map of the number of common respiratory visits in children, routine atmospheric pollutants, temperature, and relative humidity.

Figure 1 shows the correlation between the number of URTI, PNMN, BCT, and BCLT visits in children and PM_2.5_, PM_10_, O_3_, NO_2_, SO_2_, CO, temperature, and relative humidity on the same day. The correlation coefficients for relative humidity and NO_2_, relative humidity and temperature only, were not statistically significant (*p* > 0.05). The number of four common respiratory visits in children was positively correlated with PM_2.5_, PM_10_, NO_2_, SO_2_, and CO, and negatively correlated with temperature and relative humidity. Theoretically, O_3_ causes respiratory irritation in humans [39], but this paper concluded that the number of common respiratory visits in children was negatively correlated with O_3_. Hu et al. [40] obtained a correlation coefficient of −0.978 between the number of emergency admissions for respiratory infections in children and O_3_ concentration, i.e., a stronger negative correlation than that obtained in this study. This may be because the other five conventional air pollutants, which were negatively correlated with O_3_, had a greater effect on the number of respiratory visits than O_3_. Higher (lower) concentrations of the other five pollutants resulted in an increased (decreased) number of visits, while lower (higher) concentrations of O_3_ resulted in a decreased (increased) number of visits.

### 3.2. GAM of Single Pollutants

**Figure 2:**
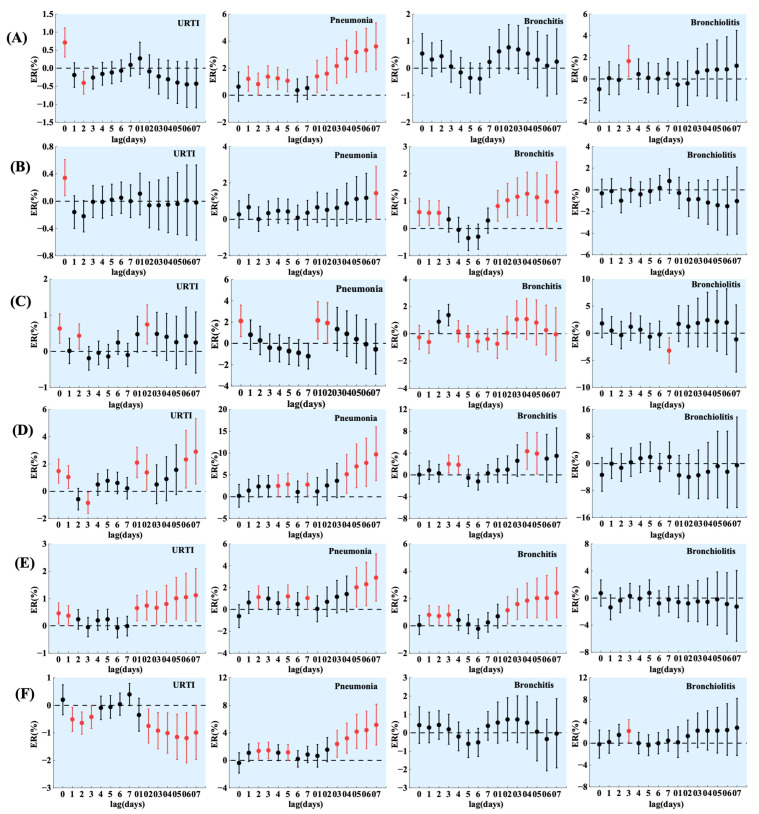
Effect of air pollutants on the number of common respiratory visits in children on different lag days. (**A**) PM_2.5_, (**B**) PM_10_, (**C**) O_3_, (**D**) NO_2_, (**E**) SO_2_, and (**F**) CO (PM_2.5_, PM_10_, NO_2_, and O_3_ increase by 10 μg/m^3^ per unit concentration. SO_2_ increases by 1 μg/m^3^ per unit concentration. CO increases by 0.1 mg/m^3^ per unit concentration. Data in red are statistically significant (*p* < 0.05).).

Introducing air pollutants and the number of URTI, PNMN, BCT, and BCLT visits into the GAM gave the ER and 95% CI shown in Figure 2. 

PM_2.5_ had the greatest effect on the number of URTI visits in children at lag 0, with a 0.71% increase in visits for every 10 μg/m^3^ increase in PM_2.5_. This result was smaller than that found by Li et al. [41] in Guangzhou (ER = 1.70%) and Nanjing (ER = 0.41%), and larger than that found in their study in Shijiazhuang (ER = 0.23%), which is consistent with the conclusion of Li et al. [41] that the less polluted the city, the greater the risk of human URTIs from PM_2.5_. During the multiple-day average lag period, the ER of PNMN visits in children per 10 μg/m^3^ increase in PM_2.5_ increased with the lag period, and the maximum ER was 3.61% at lag 07. This value is larger than that found in Song et al.’ study [36] in Shijiazhuang (ER = 0.19%). PM_2.5_ significantly affected the number of BCLT visits in children only at lag 3, with an ER of 1.65%.

PM_10_ had a significant effect on the number of URTI visits in children at lag 0 and the number of PNMN visits in children at lag 07, with the number of visits increasing by 0.34% and 1.44% for every 10 μg/m^3^ increase in PM_10_. PM_10_ had the greatest effect on the number of BCT visits at lag 07, with an ER = 1.34%. A study conducted by He et al. [42] in Guangzhou also found that PM_10_ had the greatest effect on the number of emergency department visits for BCT in children at lag 07 (ER = 5.15%).

O_3_ had the greatest effect on the number of children with a URTI at lag 02, with a 0.74% increase in the number of visits for an increase of 10 μg/m^3^. O_3_ had the greatest effect on the number of children with PNMN at lag 01, with an ER of 2.16%, and O_3_ had the greatest effect on the number of children with BCT at lag 3, with an ER of 1.36%. The maximum ER of the effect of O_3_ on the number of visits for PNMN and BCT in the study was smaller than that in He et al.’s study [42] in Guangzhou (2.66%, 1.18%).

NO_2_ had the greatest effect on the number of URTI and PNMN visits in children at lag 07. The number of visits increased by 2.90% and 9.70% per 10 μg/m^3^ increase in NO_2_, respectively. The effect of NO_2_ on the number of PNMN visits obtained in this study was greater than that found in Lanzhou’s study [35] (ER = 1.73%), which may be due to the fact that the NO_2_ pollution in Lanzhou was more severe than that in Zhengzhou during the study period [36]. The effect of NO_2_ on the number of BCT visits in children at lag 04 was the greatest, with an ER of 4.33%. This value is smaller than the maximum value of the ER of BCT emergency department visits in children per 10 μg/m^3^ increase in NO_2_ (7.22%) in Guangzhou [42].

The effects of SO_2_ on URTI, PNMN, and BCT visits in children all reached their maximum value at lag 07, with an increase of 1 μg/m^3^ in SO_2_ associated with an increase of 1.12%, 2.91%, and 2.41% in the number of visits, respectively.

The effect of CO on the number of PNMN visits was greatest at lag 07, with a 5.16% increase in visits for every 0.1 mg/m^3^ increase in CO. CO had a significant effect on the number of BCLT visits only at lag 3, with an ER of 2.24%.

The ER obtained in this study is not in full agreement with the results of existing studies in different cities, but it is basically in line with the conclusions of Li et al. [41] and Song et al. [36]. They concluded that the lighter the pollution, the higher the ER. The differences in ER may also be related to the composition of pollutants in different regions and the population’s different living habits and physical fitness.

Overall, the number of visits for four common respiratory in children was significantly affected by a variety of air pollutants. Most of the ER maxima occurred during the lag period, and the lag effects of air pollutants on the number of respiratory visits in children were significant. On the lag day with the greatest effect, the ER of URTIs and BCT was greater for NO_2_ and SO_2_ compared to other air pollutants, and the ER of PNMN was greater for NO_2_, SO_2_, and CO, and that of BCLT was greater for CO.

### 3.3. Stratified Analysis

#### 3.3.1. Sex-Stratified Analysis

Table 1 shows the effects of air pollutants on the number of common respiratory visits in children of different sexes on the day of the greatest effect. PM_2.5_ had a significant effect on the number of URTI visits in both boys and girls, with a greater effect on girls than on boys. O_3_ and NO_2_ had a significant effect on the number of URTI visits in boys and girls, respectively. The number of PNMN visits for both boys and girls was significantly affected by PM_2.5_, NO_2_, SO_2_, and CO, and the number of PNMN visits for girls was also significantly affected by O_3_. PM_10_, O_3_, NO_2_, and SO_2_ had a significant effect on the number of BCT visits for boys, but not for girls. And PM_2.5_ and CO had a significant effect on the number of BCLT visits for boys, but not for girls. Overall, there were no significant sex differences in the effects of air pollutants on the number of URTI and PNMN visits in children, with boys being more susceptible to BCT and BCLT. There were no significant sex differences in the effects of PM_2.5_, O_3_, NO_2_ and CO on the number of common respiratory visits in children. Taken together, PM_10_ and SO_2_ had a greater impact on the number of respiratory visits in boys than in girls, with Ma et al. [43] deriving a 0.06% increase in respiratory emergency admissions per 10 μg/m^3^ increase in PM_10_ in boys compared to girls. These sex differences may be due to physiological differences such as different airway thickness [35] and different activity preferences [44].

#### 3.3.2. Age-Stratified Analysis

The effects of air pollutants on the number of common respiratory visits in children of different ages on the day of the strongest effect were analyzed in Table 2. The number of BCLT visits in children aged 7–16 years was 0. The number of respiratory visits in children of different ages was affected by different air pollutants. The number of URTI visits was significantly affected by five pollutants: PM_2.5_, PM_10_, O_3_, NO_2_, and SO_2_. The number of PNMN visits in children aged 0–3 years and 7–13 years was significantly affected by air pollutants. The number of BCT visits in children aged 1–13 years was significantly affected by two pollutants. The number of BCLT visits in children younger than 1 year of age was significantly affected by PM_2.5_. In general, age differences in the impact of air pollutants on the number of different respiratory visits varied.

### 3.4. GAM of Two Pollutants

**Figure 3:**
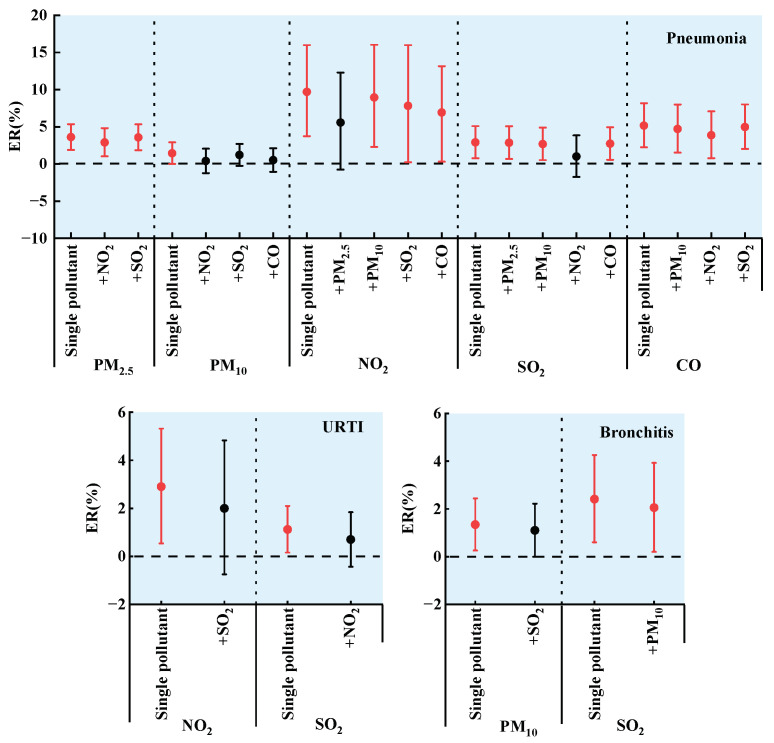
Effect of air pollutants on the number of common childhood respiratory visits in single- and two-pollutant models (PM_2.5_, PM_10_, NO_2_, and O_3_ increase per unit concentration of 10 μg/m^3^. SO_2_ increase per unit concentration of 1 μg/m^3^. CO increase per unit concentration of 0.1 mg/m^3^. Data in red are statistically significant (*p* < 0.05).).

The two air pollutants with the same strongest effect day were entered simultaneously into the GAM, and the ER and 95% CI were obtained, as shown in Figure 3. Strong covariance existed between pollutants with correlation coefficients greater than or equal to 7 [36], so PM_2.5_ and PM_10_ and PM_2.5_ and CO were not entered into the two-pollutant GAM at the same time. The effect of PM_2.5_ on the number of PNMN visits in children increased when CO was included in the model. When considering NO_2_ or SO_2_, the effect of PM_2.5_ on the number of PNMN visits in children was stable, which is consistent with the results obtained by Li et al. [41] on the effect of PM_2.5_ on the number of childhood respiratory disease visits in Xi’an after adding SO_2_. The effect of PM_10_ on the number of PNMN visits was not statistically significant after the introduction of NO_2_, SO_2_, or CO. He et al. [45] obtained similar results of a decrease in the effect of PM_10_ on the number of childhood PNMN admissions after the addition of SO_2_. The effect of NO_2_ on the number of PNMN visits in children was no longer significant after the introduction of PM_2.5_, and the effect of NO_2_ was reduced after the introduction of PM_10_, SO_2_, and CO. He et al. [45] reached a similar conclusion that the effect of NO_2_ on the number of childhood PNMN admissions was no longer significant after the introduction of PM_2.5_, PM_10_, or SO_2_ into the model. The effect of SO_2_ on the number of PNMN visits in children was stable when PM_2.5_, PM_10_, or CO was included. The effect of CO on the number of PNMN visits in children was stable when PM_10_, NO_2_, or SO_2_ was included. The effects of NO_2_ and SO_2_ on the number of URTI visits in children were no longer significant in the two-pollutant model. The ERs obtained by Zheng et al. [46] for the two-pollutant model for SO_2_ and NO_2_ were reduced by 6.66% and 4.18%, respectively, compared with the single-pollutant model. The effects of PM_10_ and SO_2_ on the number of BCT visits in children in the two-pollutant model were smaller than the effects of the pollutants on the number of visits in the single-pollutant model. He et al. [45] reached similar conclusions when they investigated the effects of PM_10_ and SO_2_ on the number of hospital admissions for BCT in children. The study found that some of the results of the two-pollutant GAM were smaller or no longer significant than the single-pollutant results. The study obtained some two-pollutant GAM results that were smaller than the single-pollutant results or no longer significant, which is similar to the findings of Liu et al. [47]. This may be due to the correlation between atmospheric pollutants interfering with the analysis results [45], and whether gaseous pollutants should be introduced into the two-pollutant model at the same time as particulate matter to study their health effects needs further research [47].

The effects of air pollutants on the number of common pediatric respiratory visits changed when other pollutants were introduced, indicating the presence of complex interactions between different air pollutants.

## 4. Conclusions

To investigate the relationship between the number of visits for URTI, PNMN, BCT and BCLT in children and the concentration of conventional air pollutants, this study first used Spearman correlation analysis to obtain the correlation coefficients between the number of visits for four pediatric respiratory diseases and the concentration of air pollutants. A generalized additive model was then used to calculate the percentage increase in the number of visits for the four respiratory diseases for each unit increase in air pollutant concentration. Additionally, we assessed the percentage variation in visits for children of varying sexes and ages. Finally, the effect of air pollutants on the number of visits for respiratory diseases was investigated in the two-pollutant model. The main conclusions are as follows:

(1) The number of visits for URTI, PNMN, BCT, and BCLT in children was positively correlated with PM_2.5_, PM_10_, NO_2,_ SO_2,_ and CO. Conversely, it was negatively correlated with temperature and relative humidity.
(2) The number of URTI, PNMN, BCT, and BCLT visits in children was significantly affected by a variety of air pollutants, with a lag effect. On the day of the strongest effect, NO_2_ and SO_2_ had a greater effect on the number of URTI and BCT visits in children; NO_2_, SO_2_, and CO had a more pronounced effect on the number of PNMN visits in children; and CO had a greater effect on the number of BCLT visits in children.
(3) The number of URTI, PNMN, BCT, and BCLT visits in children of different ages was affected differently by air pollutants. There were no significant sex differences in the number of URTI and PNMN visits in children affected by air pollutants. However, the number of BCT and BCLT visits in boys was more likely to be affected by air pollutants compared to girls.
(4) The effects of pollutants on the number of common respiratory visits in children differed between the two-pollutant GAM and a single pollutant. Additionally, there may be complex interactions between different air pollutants.

This paper examined the association between the number of URTI, PNMN, BCT, and BCLT visits in children and the concentration of air pollutants, providing a theoretical basis for air pollution control and respiratory health protection in children. The effects of environmental pollution on human health have not been very comprehensively investigated yet, so any attempt in this difficult-to-study area is highly urgent. Furthermore, studies have shown that formaldehyde and other unregulated pollutants can also affect human respiratory morbidity. Therefore, further research will be conducted on the correlation between formaldehyde and respiratory diseases in children. Secondly, the city studied was located in specific regions, which may affect the generalizability of the results. Future research needs to use more data over a wider area to validate the findings.

## Figures and Tables

**Table 1:** Effect of air pollutants on the number of common respiratory visits in children of different sexes.

		ER (%) and 95% CI					
		PM_2.5_	PM_10_	O_3_	NO_2_	SO_2_	CO
URTI	Boy	**0.69** **(0.15, 1.22)**	0.32 (−0.03, 0.67)	**0.84** **(0.13, 1.554)**	0.42 (−2.59, 3.53)	0.99 (−0.28, 2.27)	—
	Girl	**0.73** **(0.12, 1.34)**	0.35 (−0.05, 0.76)	0.67 (−0.15, 1.50)	**6.24** **(2.59, 10.01)**	1.37 (−0.05, 2.82)	—
PNMN	Boy	**3.42** **(1.18, 5.71)**	1.30 (−0.58, 3.21)	1.78 (−0.43, 4.03)	**7.67** **(0.16, 15.74)**	**3.93** **(1.11, 6.83)**	**4.23** **(0.46, 8.15)**
	Girl	**3.92** **(1.32, 6.58)**	1.33 (−0.82, 3.52)	**2.89** **(0.12, 5.74)**	**13.29** **(4.53, 22.78)**	**3.28** **(0.15, 6.51)**	**6.58** **(2.10, 11.26)**
BCT	Boy	—	**2.23** **(0.86, 3.63)**	**1.44** **(0.48, 2.41)**	**5.50** **(1.35, 9.81)**	**3.89** **(1.66, 6.17)**	—
	Girl	—	0.33 (−1.35, 2.03)	1.26 (−0.06, 2.60)	3.03 (−2.01, 8.34)	0.98 (−1.77, 3.81)	—
BCLT	Boy	**2.13** **(0.37, 3.93)**	—	—	—	—	**2.68** **(0.11, 5.31)**
	Girl	0.81 (−1.50, 3.17)	—	—	—	—	1.39 (−1.91, 4.81)

Note: PM_2.5_, PM_10_, NO_2_, and O_3_ increase per unit concentration of 10 μg/m^3^; SO_2_ increase per unit concentration of 1 μg/m^3^; and CO increase per unit concentration of 0.1 mg/m^3^. Bolded data are statistically significant (*p* < 0.05).

**Table 2:** Effect of air pollutants on the number of common respiratory visits in children of different ages.

		ER (%) and 95% CI					
		PM_2.5_	PM_10_	O_3_	NO_2_	SO_2_	CO
URTI	<1	0.75 (−0.12, 1.63)	**0.57** **(0.01, 1.13)**	−0.14 (−1.21, 0.93)	1.95 (−2.48, 6.58)	0.76 (−1.15, 2.70)	—
	1–3	**0.76** **(0.17, 1.35)**	0.32 (−0.06, 0.71)	0.46 (−0.32, 1.26)	1.70 (−1.63, 5.15)	0.99 (−0.43, 2.42)	—
	4–6	0.33 (−0.54, 1.21)	−0.09 (−0.69, 0.51)	**2.65** **(1.35, 3.97)**	4.13 (−1.44, 10.01)	0.43 (−1.63, 2.54)	—
	7–13	**1.59** **(0.20, 3.00)**	0.80 (−0.08, 1.69)	**2.76** **(0.87, 4.69)**	**10.60** **(2.43, 19.42)**	**4.05** **(0.79, 7.42)**	—
	14–16	3.36 (−1.42, 8.37)	**3.55** **(0.50, 6.68)**	−3.34 (−10.07, 3.90)	−14.60 (−34.19, 10.82)	−6.18 (−16.05, 4.84)	—
PNMN	<1	**3.61** **(0.86, 6.44)**	1.91 (−0.38, 4.25)	1.02 (−1.61, 3.72)	8.28 (−1.00, 18.42)	3.15 (−0.33, 6.76)	**7.15** **(2.52, 11.99)**
	1–3	**3.67** **(1.16, 6.24)**	1.79 (−0.34, 3.96)	**3.50** **(0.77, 6.30)**	**10.93** **(2.14, 20.49)**	**3.50** **(0.29, 6.82)**	3.81 (−0.47, 8.26)
	4–6	4.14 (−0.60, 9.10)	−0.44 (−4.28, 3.55)	2.13 (−2.78, 7.27)	3.23 (−10.87, 19.54)	5.69 (−0.03, 11.74)	5.85 (−2.48, 14.88)
	7–13	2.71 (−6.48, 12.80)	−3.82 (−10.18, 2.98)	6.34 (−0.29, 13.42)	**31.34** **(1.88, 69.32)**	7.78 (−2.20, 18.78)	3.36 (−10.55, 19.43)
	14–16	2.13 (−28.86, 46.61)	−9.12 (−35.29, 27.65)	0.32 (−32.90, 49.98)	−17.23 (−75.27, 177.10)	−20.05 (−50.90, 30.19)	1.94 (−44.20, 86.24)
BCT	<1	—	2.11 (−0.08, 4.36)	0.74 (−0.96,2.47)	**8.45** **(1.59,15.77)**	0.95 (−2.52,4.54)	—
	1–3	—	1.23 (−0.17, 2.65)	**1.42** **(0.41, 2.44)**	1.90 (−2.23, 6.19)	**2.62** **(0.29, 5.00)**	—
	4–6	—	1.37 (−1.08, 3.88)	1.69 (−0.22, 3.63)	**10.95** **(3.81, 18.58)**	**7.40** **(4.11, 10.79)**	—
	7–13	—	**7.42** **(0.02, 15.37)**	1.82 (−2.10, 5.89)	11.02 (−9.06, 35.54)	**16.81** **(4.35, 30.77)**	—
	14–16	—	The confidence interval is wide.			−10.90 (−46.57, 48.60)	
BCLT	<1	**1.89** **(0.27, 3.54)**	—	—	—	—	2.15 (−0.12, 4.48)
	1–3	0.71 (−1.71, 3.20)	—	—	—	—	1.24 (−2.25, 4.85)
	4–6	2.21 (−24.96, 39.23)	—	—	—	—	−2.75 (−36.59, 49.17)

Note: PM_2.5_, PM_10_, NO_2_, and O_3_ increase per unit concentration of 10 μg/m^3^; SO_2_ increase per unit concentration of 1 μg/m^3^; and CO increase per unit concentration of 0.1 mg/m^3^. Bolded data are statistically significant (*p* < 0.05).

## Data Availability

Not applicable.
